# What distinguishes patients with mass social media-induced illness presenting with Tourette-like behavior from those with Tourette syndrome? Results of a prospective cohort study

**DOI:** 10.1007/s00406-023-01603-z

**Published:** 2023-05-20

**Authors:** Carolin Fremer, Natalia Szejko, Anna Pisarenko, Martina Haas, Luise Laudenbach, Claudia Wegener, Kirsten R. Müller-Vahl

**Affiliations:** 1 Clinic of Psychiatry, Social Psychiatry and Psychotherapy, Hannover Medical School, Carl-Neuberg-Straße 1, 30625 Hannover, Germany; 2 Department of Neurology, Medical University of Warsaw, Warsaw, Poland; 3 Department of Bioethics, Medical University of Warsaw, Warsaw, Poland; 4 Department of Audiovisual Media Studies, Film University Babelsberg, Potsdam, Germany

**Keywords:** Tourette-like behavior, Social media, Mass sociogenic illness, Functional movement disorders, Tourette syndrome

## Abstract

Since 2019, a global increase in patients presenting with functional Tourette-like behaviors (FTB) has been observed. This has been related to the exposure of tic-related content in social media, although other factors seem to further fuel this phenomenon. Recently, we, therefore, proposed the term mass social media-induced illness (MSMI) as, in our opinion, this phenomenon constitutes a new type of mass sociogenic illness (MSI) that is in contrast to all recent outbreaks spread solely via social media. In accordance with this hypothesis, we were able to identify the host of the German YouTube channel "Gewitter im Kopf" (“Thunderstorm in the brain”) as the initial virtual index case. The purpose of this paper is to present clinical characteristics of a sample of 32 patients diagnosed with MSMI-FTB compared to a large sample of patients with Tourette syndrome (TS) and other chronic tic disorders (CTD) (*n* = 1032) from the same center in Germany indicating clinical factors helpful to distinguish between tics in TS/CTD and MSMI-FTB. Our main findings were: in patients with MSMI-FTB compared to those with TS/CTD we found (i) a significantly higher age at onset, (ii) a significantly higher rate of females, (iii) a significantly higher rate of obscene and socially inappropriate symptoms, (iv) a significantly lower rate of comorbid ADHD, and (v) a significantly lower rate of OCD/OCB. In contrast, rates of comorbid anxiety and depression as well as reported frequencies of premonitory urges/sensations and suppressibility of symptoms did not differ between groups.

## Introduction

Starting in spring 2019, in Germany an outbreak of functional Tourette-like behaviors (FTB) has been observed [1]. Soon thereafter it became clear that patients with FTB with very similar symptomatology are presenting in specialist Tourette centers in many countries worldwide [2–11]. Today, there is general agreement among experts that this outbreak is closely related to popular videos shown on social media platforms such as YouTube and TikTok [8, 9]. In Germany, the German-speaking YouTube channel "Gewitter im Kopf" (“Thunderstorm in the brain”) could be identified as most influential because of a remarkable symptom overlap between patients and the channel host [1]. Claiming to suffer from extreme Tourette syndrome (TS), in his videos he purports to educate about this disorder [12]. In English-speaking countries however other influencers have been identified as most influential such as the British Evie Meg (“thistrippyhippie”) and [13] the Danish Stine Sara [14].

Based on recent literature, a clear symptom overlap between individuals affected is obvious with relatively abrupt onset of mainly complex “tic-like” symptoms often accompanied by socially inappropriate behavior in patients at teenage age [2–11]. Interestingly, with some minor country-specific differences, not only the patients affected, but also social media influencers show remarkable similarities presenting mainly with complex, stereotyped movements at arms and body and vocalizations dominated by swear words, comments, and insults [1–11].

Contrary to FTB, tics are rapid, repetitive, non-rhythmic movements or vocalizations [15]. They can be divided into motor vs. vocal and simple vs. complex tics. Motor tics follow a typical and very characteristic rostro-caudal distribution. Since TS is regarded as a neurodevelopmental disorder, tics start on average at the age of 5–7 years [16]. The onset is slow most typically with simple motor tics such as eye blinking, grimacing, or head jerks followed by simple vocal tics such as throat clearing on average 2 years later [17]. Peak tic severity is reached in most patients at the age of 10 to 12 years [18]. In the majority of patients, comorbid conditions occur such as obsessive–compulsive behavior/disorder (OCB/OCD), attention deficit/hyperactivity disorder (ADHD), depression, and anxiety. For still unknown reasons, males are 3–4 times more commonly affected than females [19].

Only recently, we presented for the first time a more in-depth characterization of a large cohort of patients with mass social media-induced illness (MSMI) [20] as a new type of mass sociogenic illness (MSI) spread only via social media. Unlike former “classical” outbreaks of MSI dating way back in history [21], for MSMI outbreaks face to face contact between individuals is not necessary, since they are centered around a virtual index case. In our recent study [20], we identified timely-related psychological stressors, unconscious intrapsychic conflicts, and/or structural deficits in all patients suggesting these parameters as predisposing factors for MSMI-FTB. In addition, the majority of patients suffered from further psychiatric symptoms, most commonly OCB, anxiety, depression, and problems with social interactions. In line with recent reports [6, 10], in almost half of the patients pre-existing TS was diagnosed suggesting the presence of tics as another independent predisposing factor for the development of MSMI that may explain the specific phenotype of the current outbreak presenting with FTB.

Because of some similarities between tics in TS and FTB, it is of utmost importance to identify factors that enable differentiation between these conditions, since both diagnoses still have to be made merely clinically. Moreover, symptoms require different treatment approaches, which can only be applied after a correct diagnosis has been made to alleviate the usually high impairment of those affected. According to the results of a poll performed among TS experts conducted at the 14th European Conference on Tourette Syndrome & Tic Disorders in June 2022 by the European Society for the Study of Tourette Syndrome (ESSTS), only 50% of TS experts have confidence being able to differentiate tics/TS from FTB – even after having attended lectures on this topic (unpublished data). Thus, for general neurologists and psychiatrists the differential diagnosis may be even more challenging. Accordingly, in 2021, a first study was published based on a very small German sample revealing the following differences between tics/TS and FTB: type and age of onset, symptom fluctuation and deterioration, complexity of symptoms, sex proportion, comorbidities, medical treatment, socially inappropriate behaviors/coprophenomena, self-injurious behaviors, hospitalization, and school absenteeism [10]. Thereafter, further studies aiming to identify differences between TS and (MSMI-)FTB have been published, but all of these are limited by either rather small sample sizes (FTB: number of patients ranging from 9 to 22, chronic tic disorder (CTD)/TS: numbers of patients between 13 to 270), the inclusion of patients with FTB with no clear relationship to social media use, or utilization of only very few clinical items [5, 6, 10, 11, 22].

Here, we present clinical characteristics of a sample of 32 patients diagnosed with MSMI-FTB compared to a large sample of patients with TS and other CTD (*n* = 1032) from the same center indicating clinical factors helpful to distinguish between tics/TS and MSMI-FTB. Our second goal was to formulate clues that could help clinicians in the differential diagnosis between these two disorders. We hypothesized that patients with MSMI-FTB differ in many ways from patients with TS such as later age of symptoms’ onset, female preponderance, atypical or absent premonitory sensations, and higher prevalence of obscene and socially inappropriate symptoms.

## Methods

Between 5/2019 and 9/2021, 32 patients (mean age: 20.1 years, range: 11–53 years, median age: 18 years, *n* = 16 females (50%)) diagnosed with MSMI-FTB at our Tourette outpatient clinic agreed to participate. All patients underwent a neuropsychiatric examination by a neurologist and psychiatrist with more than 20 years of clinical experience with patients with tics (KMV) who confirmed the diagnosis. Using a detailed semi-structured clinical and psychological interview composed by the authors and conducted by a psychologist with extensive experience with TS (CF), we took record of demographic data such as sex, age at evaluation, and age at symptom onset. In addition, a detailed history was taken of all new-onset symptoms in terms of suppressibility, premonitory sensations, socially inappropriate behaviors including insults, swear words, and other socially inappropriate or obscene behaviors as well as comorbid psychiatric symptoms. All current and recent symptoms reported by patients, parents, and observed during the examination were documented. Interviews were conducted together with the parents, depending on the age and whether patients were able to report in detail themselves. For further details regarding the study protocol please refer to [20]. Characteristics in patients with MSMI-FTB were compared to a large sample of patients with TS/CTD (*n* = 1032 including 529 children, mean age: 20.9 years, range: 4–72 years, sex ratio m/f: 3.4:1). For further details regarding this sample please refer to [23]. The study was reviewed and approved by Local Ethics Committee at Hannover Medical School (No. 8995_BO_S_2020).

### Statistical analyses

Using Excel (Microsoft Office Professional Plus 2016 version) frequencies, means, medians, and distribution measures were calculated. R (version 4.1.1) was used for conducting analyses for group comparisons [24]. *T*-tests were performed for interval-scaled dependent variables within group comparisons and Fisher's exact tests for dichotomous dependent variables. For group comparisons, *p*-values of significant results were conservatively corrected for multiple comparisons according to Bonferroni (*p*_*c*_). Additionally, as measures of the effect size of significant results Cohen's d and the bias-corrected Cramer's V were calculated.

## Results

### General characteristics

For statistical analyses we assigned patients to their biological sex, while exactly half of our sample of 32 included patients was female (*n* = 16; 50%) giving a ratio of 1:1. On average patients were 20.1 years (range: 11–53 years, median age: 18 years) old at time of evaluation. On average FTB started 11 months earlier at the age of 19.2 years (range: 10–53 years, median age: 17 years). Compared to patients with TS/CTD (*n* = 1032), in patients with MSMI-FTB we found (i) a significantly higher age at onset (with a large effect) (*t*(1062) = 18.63, *p*_*c*_ < 0.001, *d* = 2.53) and (ii) a significantly higher rate of females (with a small effect) (*p*_*c*_ = 0.01, *V* = 0.11). No significant differences were found with respect to age at evaluation (*p*_*c*_ = 1).

### Clinical characteristics of FTB and comorbidities

When comparing for clinical characteristics, in patients with MSMI-FTB we found a significantly higher rate of obscene words and other socially inappropriate symptoms (with a medium effect) (*p*_*c*_ < 0.001, *V* = 26). No significant differences were found with respect to reported frequencies of premonitory urges/sensations (*p*_*c*_ = 0.530) and suppressibility of tics and MSMI-FTB, respectively (*p*_*c*_ = 1) (if assessed as dichotomous variables (yes/no)).

With regard to comorbidities we found (i) a significantly lower rate of comorbid ADHD (with a small effect) (*p*_*c*_ = 0.003, *V* = 12) and (ii) a significantly lower rate of OCD/OCB (with a small effect) (*p*_*c*_ = 0.046, *V* = 0.09) in patients with MSMI-FTB. No significant differences were found with respect to presence of comorbid anxiety (*p*_*c*_ = 1) and depression (*p*_*c*_ = 1). For further details see Table 1.

**Table 1:** Comparison between patients with mass social media-induced illness presenting with functional Tourette-like behavior (MSMI-FTB) (*n* = 32) and a large sample of patients with Tourette syndrome (TS) or other chronic tic disorders (CTD) (*n* = 1032)

	FTB (*n* = 32)	TS/CTD (*n* = 1032)	*p*^a,c^	*p*_*c*_^b,c^	Effect size
Age at evaluation (year, mean ± *SD*)	20.1 ± 11.0	20.9 ± 12.9	0.686	1	-
Age at onset (year, mean ± *SD*)	19.2 ± 11.01	7.0 ± 3.2	< **0.001**	< **0.001**	*d* = 2.53
Sex ratio (male:female)	1:1	3.4:1	**0.001**	**0.01**	*V* = 0.11
*Clinical characteristics*					
Premonitory sensation/urge (*n*, %)	27 (84.4%)	700 (67.8%)	0.053	0.53	*-*
Suppressibility (*n*, %)	27 (84.4%)	853 (85.4%)	1	1	*-*
Obscene and socially inappropriate symptoms/coprophenomena (*n*, %)	31 (96.9%)	290 (28.1%)	< **0.001**	< **0.001**	*V* = 21
*Comorbidities*					
ADHD (*n*, %)	3 (9.0%)	463 (44.9%)	< **0.001**	**0.003**	*V* = 12
OCB/OCD (*n*, %)	15 (46.9%)	740 (71.8%)	**0.005**	**0.046**	*V* = 0.09
Anxiety (*n*, %)	13 (40.6%)	323 (31.4%)	0.334	1	-
Depression (*n*, %)	10 (31.3%)	236 (22.9%)	0.287	1	-

*FTB* functional tic-like behaviors, *TS *Tourette syndrome, *CTD* chronic tic disorder, *SD* standard deviation, *ADHD* attention deficit/hyperactivity disorder, *OCB* obsessive compulsive behavior, *OCD *obsessive–compulsive disorder, *d* Cohen’s d, *V* bias-corrected Cramer’s V

^a^Uncorrected *p*-values

^b^Corrected *p*-values according to Bonferroni

^c^*t*-tests were performed for interval-scaled dependent variables and Fisher's exact tests for dichotomous dependent variables; significant results are shown in bold (*p* < 0.05)

## Discussion

In this study, we further elaborated differences in clinical characteristics between MSMI-FTB and tics in patients with TS/CTD. Based on our data and in contrast to tics, MSMI-FTB is characterized by (i) an onset of symptoms at teeange age or in young adulthood and thus several years after typical onset of tics in preschool age, (ii) a higher rate of females, (iii) a much higher occurrence of socially inappropriate and obscene behaviors compared to coprophenomena in TS/CTD; (iv) a lower prevalence of comorbid ADHD, and (v) a lower prevalence of OCD/OCB compared to TS/CTD. In line with recent work [5, 6, 8, 10], we were not able to identify *one* single factor that allows a clear differentiation between (MSMI-)FTB and tics/TS/CTD. Similarly to Paulus et al. [10] and Pringsheim et al. [5, 6], we detected no differences with respect to reported frequencies of premonitory urges/sensations and suppressabilty of symptoms. Finally, we found no differences in frequencies of anxiety and depression, which were common comorbidities in both groups of patients.

So far, only four other studies have been published comparing directly clinical characteristics of patients with MSMI-FTB and tics/TS [5, 6, 10, 11]. All these studies, however, are limited by very small sample sizes. Interestingly, in the only other German study including only 13 patients in each patient group, similar results have been reported with respect to age at onset [10]. Different to our study, however, in this study no differences were found concerning prevalance rates of comorbidites including ADHD and OCD as well as frequencies of socially inappropriate behavior/coprophenomena, premonitory sensations, and suppressability, which might possibly be related to the small sample size. Similar to our study (male to female ratio = 1:1), a relatively large proportion of patients were males (62%), while in all other studies [5, 6, 11], there was a definite predominance of females. It has been speculated that this is due to the fact that in Germany the (virtual) index case relevant for the MSMI outbreak was male, while in all other regions of the world presumable index cases were female [20].

Compared to the other studies outside of Germany [5, 6], in patients with FTB compared to those with TS the following differences were found: younger age at time of investigation, but older age at onset, predominance of females, higher severity scores of motor and vocal symptoms, higher impairment scores, more complex arm/hand motor movements, more complex vocalizations, more obscene words, higher scores on self-report measures for ADHD, OCD, anxiety, and depression as well as more frequent diagnosis of depression. These discrepancies might be related to methodological factors such as partly retrospective data collection, small sample sizes, different male to female ratios, inclusion of adult versus pediatric or mixed populations, and different measurements. In future studies, therefore, specific attention should be drawn to psychiatric comorbidities using adequate instruments.

In line with all recent reports [5, 6, 10, 11], we found in patients with MSMI-FTB compared to patients with TS/CTD a much higher frequency of complex vocalization, obscene words, and socially inappropriate and obscene behaviors. Remarkably, in line with recent reports [5, 6, 10] we found no differences in patients with MSMI-FTB compared to those with TS/CTD with respect to reported frequencies of premonitory urges/sensations and suppressibility of symptoms. Based on older studies in patients with “functional tics” (without any influence from social media) [25–28], it was believed that “functional tics” are only rarely accompanied by premonitory sensations and can typically not be suppressed voluntarily. In contrast, these two factors have been considered as hallmarks of tics [23]. Based on our data, patients’ reports on the *prevalence* of premonitory sensations and suppressibility cannot be regarded as reliable factors that can be used for differentiation of MSMI-FTB from tics. However, in a recent study from our group, we were able to demonstrate that instead the *kind* of descriptions of premonitory sensations and supressibility including duration, characteristics, and influencing factors largely differs in patients with MSMI-FTB compared to those in patients with TS and can reliably be used for differentitaion between these two phenomena [20].

Because of the disparities between tics and MSMI-FTB, we suggest not to use the term “tics” when reporting and describing FTB. In our opinion this is of particular importance in those patients who suffer from both conditions [20]. Furthermore, from a historical perspective, for many years it was believed that tics in TS represent a functional (“psychogenic”) symptom [29] and therefore a clear distinction between tics and FTB should also be expressed in the terminology used. Since the vast majority of patients with MSMI-FTB presents with movements, vocalizations, and socially inappropriate behaviors, which also stays in line with common opinion about TS in general population, we suggest to use the term “Tourette-like” instead of “tic-like” behavior since it encompasses all these symptoms.

The following limitations of our study have to be considered: (i) a selection bias cannot be excluded as probably only patients with more severe phenotypes presented to our center; (ii) although this is the largest sample comparing clinical characteristics of MSMI-FTB with TS so far, the sample size of patients with MSMI-FTB was still relatively small; (iii) our TS/CTD sample was very large and included more than 1000 patients. However, this sample was collected over a long time period several years ago and was not collected prospectively to be used as a control group for this study. Therefore, we were not able to compare further clinical features of MSMI-FTB such as type of symptom onset since there is no record of these characteristics in our TS/CTD sample; and (iv) due to lack of information on prevalence rates separately for OCD and OCB in the MSMI-FTB sample, we were able to compare only combined numbers for OCD/OCB in both groups. Since the prevalence of 72% of OCD/OCB in our TS/CTD sample can be considered as representative [30–34], different frequencies can be regarded as robust data and thus clinically relevant.

In addition, the following strengths of our studies can be highlighted: firstly, we used a prospective design for data collection of the MSMI-FTB sample; secondly, in both samples, TS/CTD and MSMI-FTB, we included both children and adults, while in most other studies mainly children and teenagers had been included [3, 4, 6, 8]; and thirdly, while in this study, we included only patients, where both a clear temporal link between symptom onset and use of the YouTube channel “Gewitter im Kopf” could be demonstrated and in addition a clear symptom overlap between patients and the channel host was obvious, in most other studies, patients with FTB had been included even in the absence of such a clear connection. Thus, it can be speculated that reported samples may differ slightly, since in the same time period we saw in addition to patients with MSMI-FTB several further patients with FTB, but without this obvious interrelationship with social media. Those further patients presented either with the „new variant” of FTB with rapid onset and mainly complex movements and vocalizations but without a clear connection to the YouTube channel „Gewitter im Kopf” or with the more “classic variant” of FTB as seen for many years and described earlier [26–28, 35, 36]. Based on our clinical experience and in line with the literature [20, 37], patients with the “classic variant” of FTB show no or much less swear words, insults, and comments, no or much less socially inappropriate behaviors, an overall much lower number of movements and vocalizations, and much less influential and triggering factors, but a higher number and more severe self-injurious behaviors.

Overall, this study has shown in line with other studies that there are distinct features to differentiate MSMI-FTB from tics/TS/CTD. Special attention should be paid to the kind and number of obscene words and other socially inappropriate behaviors compared to coprophenomena in TS/CTD. Other important distinguishing factors are later age at onset and larger number of females affected. Although the pattern on psychiatric comorbidities may differ, based on current data this cannot be used for differentiation. Finally, premonitory sensations and suppressibility are reported in similar frequencies in MSMI-FTB compared to TS/CTD, and the kind of descriptions largely differs.

## Data Availability

The data that support the findings of this study are available within the paper. Further inquiries can be directed to the corresponding author.
